# Therapeutic Notes

**Published:** 1895-03-02

**Authors:** 


### THERAPEUTIC NOTES.

### PERMANGANATE OF POTASSIUM AS AN ANTIDOTE IN POISONING BY MORPHINE AND OTHER SUBSTANCES.

Dr. W. Moor[1] has shown that permanganate of potassium is an efficient antidote in morphine poisoning, when both bodies are taken by the stomach. The potassium permanganate, to act well, must be given immediately after the morphine, and come into contact with it in the viscus. Dr. Moor, however, is of opinion that it may be valuable even in late stages, as it has been shown that morphine is largely excreted from the blood into the stomach, and hence may be acted upon during its excretion, and its reabsorption thereby prevented. After various preliminary experiments with smaller doses, Dr. Moor swallowed three grains of morphine sulphate, followed in thirty seconds by four grains of potassium permanganate in watery solution. He remained quite unaffected by the morphine, although ordinarily very susceptible to its action. From various test-tube experiments, as well as from the above results, he concludes that the morphine becomes oxidised to an inert substance. Cocaine, atropine, aconitine, veratrine, and pilocarpine failed to reduce the permanganate solution in the same way as morphine, while strychnine only did so partially.

Anthal[2] had previously found, that the administration of a ½ per cent. solution of permanganate of potassium to animals which had previously received by the mouth a poisonous dose of muscarine, strychnine, colchicum, oil of savin, or oxalic acid prevented their death, while it has long been known that the permanganate, when applied locally and thoroughly in good time, is a most efficient antidote in snake poisoning. Lately, several cases of opium and morphine poisoning have been recorded in American journals, in which the permanganate has been administered hypodermically, this treatment being followed by recovery. Some of Dr. Moor's countrymen seem to have failed to grasp the principle involved in his treatment, which is that the permanganate is a chemical antidote, and not a physiological antagonist of the morphine. Dr. Callender[3] records a case in which a boy seven years old received by mistake two drachms of laudanum, and three hours later was profoundly narcotised, with pallid face and almost imperceptible pulse, respiration slow, but not sterterous. In about two hours he gave the patient fifteen grains of permanganate of potasium hypodermically, and satisfactory recovery took place. Whiskey and warmth were also used. Drs. Gregg and Moreland[4] also relate a case of recovery in a man after two and a half ounces of laudanum, where twelve fluid drachms of a half-saturated solution of potassium permanganate were injected under the skin. Atropine and other treatment was also employed, which lessens the value of their evidence. It is difficult to believe that permanganate of potassium given hypodermically could in any way affect the action of morphine taken by the mouth, and hence, in spite of these and other reported cases, one is rather inclined to conclude that the recovery was due to the general measures employed rather than to the permanganate.

[1] N. Y. Medical Record, 1894, p. 200. [2] Pesth Med. Chir. Presse, 1893. [3] Med. Record, Sept., 1894. [4] Med. News., Philad., May, 1894.

